# Overcoming the Low Relaxivity of Gadofosveset at High Field with Spin Locking

**DOI:** 10.1002/mrm.23316

**Published:** 2011-12-09

**Authors:** O C Richardson, M L J Scott, S F Tanner, J C Waterton, D L Buckley

**Affiliations:** 1 Division of Medical Physics, University of LeedsLeeds, UK; 2 Imaging, Personalised Healthcare and BiomarkersAstraZeneca, Macclesfield, Cheshire, United Kingdom

**Keywords:** gadofosveset, *T*_1ρ_, spin locking, relaxivity

## Abstract

The contrast agent gadofosveset, which binds reversibly to serum albumin, has a high longitudinal relaxivity at lower magnetic fields (≤3.0 T) but a much lower relaxivity at high fields. Spin locking is sensitive to macromolecular content; it is hypothesized that combining this technique with the albumin-binding properties of gadofosveset may enable increased relaxivity at high fields. In vitro measurements at 4.7 T found significantly higher spin-lock relaxation rates, *R*_1ρ_ (1/*T*_1ρ_), when gadofosveset was serum albumin-bound than when unbound. *R*_1ρ_ values for a nonbinding contrast agent (gadopentetate dimeglumine) in serum albumin were similar to those for unbound gadofosveset. *R*_2_ (1/*T*_2_) values were also significantly higher at 4.7 T for serum albumin-bound gadofosveset than for unbound. Spin locking at high field generates significantly higher relaxation rates for gadofosveset than conventional contrast agents and may provide a method for differentiating free and bound molecules at these field strengths. Magn Reson Med, 2012. © 2011 Wiley Periodicals, Inc.

The clinically approved contrast agent gadofosveset trisodium (Ablavar, Lantheus Medical Imaging, N Billerica, MA, previously marketed as Vasovist, Schering AG, Germany) is a small-molecule gadolinium (Gd) chelate that acquires macromolecular properties on binding to serum albumin (SA). The gadofosveset molecule consists of a stable gadopentetate core with a phosphodiester linkage to a lipophilic albumin-binding group (1). Over 90% of the agent binds at low concentrations in human serum (2), with a lower binding fraction observed at higher gadofosveset concentrations (3) and in other species (4). The binding process increases the effective molecular weight of gadofosveset from 957 Da to 68 kDa (5), reducing its extravasation rate and prolonging its excretion time, making the agent well suited to angiography (6).

The large bound molecule has a lower tumbling rate and longer rotational correlation time (3), which facilitates a substantially (up to 10-fold) higher longitudinal relaxivity at low magnetic field strengths (4). The longitudinal relaxivity of the bound gadofosveset molecule (*r*_1bound_) peaks at around 0.5 T and decreases rapidly with increasing field strengths (7). The longitudinal relaxivity of the free (unbound) gadofosveset molecule (*r*_1free_) is slightly higher than conventional small-molecule Gd-based agents such as gadopentetate and shows only a moderate decrease with field strength (8).

The observed longitudinal relaxivity of gadofosveset (*r*_1obs_) includes contributions from both the bound and free molecules and is influenced by binding fraction and field strength. The nonlinear relationship between *r*_1obs_ and gadofosveset concentration at low field strengths can be modeled (7), although a linear approximation may suffice at low concentrations (8). At 3 T, *r*_1obs_ in plasma is still over twice that of most other clinically approved Gd-based agents (8); at higher fields, *r*_1bound_ and *r*_1free_ converge (9) to give a value of *r*_1obs_ similar to conventional Gd-based agents. Regardless of the reduction in relaxivity, the unique kinetic properties of gadofosveset resulting from its binding to albumin are displayed at all field strengths. An alternative contrast mechanism that provides high gadofosveset relaxivity at high fields may be required to fully exploit these properties.

Spin locking (SL), first described as an imaging technique in 1985 (10) but investigated in NMR prior to this (11), involves the application of a 90° excitation pulse followed by an RF pulse (phase shifted by 90° to the excitation pulse), applied for a duration of time (spin-lock time, TSL), which locks the spins in the rotating frame of reference. Relaxation of the magnetization in the presence of this SL field (*B*_1L_) is characterized by the time constant *T*_1ρ_. The SL pulse may be followed by a 90° pulse (phase shifted by 180° to the original excitation pulse) and an imaging sequence, or a 180° pulse and readout (12). *T*_1ρ_ is influenced by the strength of the SL field, which is commonly in the μT (low kHz) range, rather than the main magnetic field (*B*_0_). As a result, the image contrast generated by SL is equivalent to image contrast obtained at low magnetic fields, with the advantage that a high signal-to-noise ratio may be maintained (13). It should be noted that the SL RF pulse may contribute significantly to patient-specific absorption rate (SAR), particularly at high *B*_0_ as SAR is proportional to the product of *B*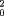, *B*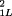 and the ratio of TSL to TR (10).

The interaction times associated with SL at very low field strengths give this technique an increased sensitivity to proteins and other macromolecules (14). This correlation between signal intensity (SI) and tissue protein has been utilized as a potential biomarker for response to tumor therapy, including treatment designed to reduce protein synthesis (15) and gene therapy resulting in reduced protein content due to cell death (16). The clinical potential of SL has also been highlighted in a study of injured myocardium (17) and the assessment of brain plaque composition in early-onset Alzheimer's disease (18).

Small-molecule Gd-based contrast agents have been used in combination with SL to provide improved myocardium–blood contrast (17, 19) and in the assessment of articular cartilage (20). SL after injection of gadopentetate has also been shown to improve tumor contrast in patients with glioma (14). A literature search (August 2011) found no published studies assessing the effect of gadofosveset on *T*_1ρ_.

The purpose of this study was to determine the potential impact of albumin binding on *T*_1ρ_ for in vitro gadofosveset solutions at a high *B*_0_ field strength. It was hypothesized that by combining the macromolecular sensitivity of SL with the albumin-binding affinity of gadofosveset a large contrast shift may be achieved at field strengths where the *T*_1_ effects of gadofosveset are very similar to those of conventional Gd-based agents. In addition, it may be possible to use this technique to generate a measurable difference in the relaxation properties of bound and unbound agent.

Tissue *T*_1ρ_ values fall between *T*_1_ and *T*_2_, with *T*_1ρ_ → *T*_2_ as *B*_1L_ → 0 (21). Conventionally, *B*_1L_ ≪ *B*_0_ therefore *T*_1ρ_ may be expected to be close to *T*_2_. As the transverse relaxivity of bound gadofosveset is known to remain high at all relevant field strengths (9), and as *T*_2_ values are known to be sensitive to tissue macromolecules (22), a further aspect of the study was to investigate whether the potential benefits of *T*_1ρ_ contrast could also be achieved using *T*_2_ contrast.

## METHODS

### Solutions

In vitro solutions of gadofosveset (Vasovist) were prepared using phosphate-buffered saline (PBS, dry powder reconstituted with deionized water, pH = 7.4, Sigma Aldrich, St Louis, MO), to replicate the unbound state, and bovine SA (BSA, Cohn fraction V lyophilized powder, Sigma Aldrich, 4.5% w/v in PBS), to replicate a combination of bound and unbound molecules similar to that found in humans. Stock solutions were created at a gadofosveset concentration of 10 mM, and serially diluted to produce further solutions at 5.0, 3.0, 2.0, 1.5, 1.0, 0.75, 0.5, and 0.25 mM. Solutions of PBS and BSA without contrast agent (0 mM) were also assessed. In order to separate the influence of the macromolecular solution from that of Gd, an equivalent set of solutions of the nonbinding contrast agent gadopentetate dimeglumine (Magnevist, Bayer Healthcare Pharmaceuticals, Germany) in BSA (4.5% w/v) were also created using the same method. Prior to scanning at 4.7 T, all solutions were heated to 37°C in a water bath; this temperature was maintained during scanning with warm air flow and verified with a fiber optic temperature probe in an adjacent water tube. At 0.5 T, samples were heated to 37°C and the temperature monitored with an integral heating system.

### Data Acquisition: *T*_1ρ_

Tubes were placed vertically in a cylindrical cradle of diameter 60 mm and inserted into a 63 mm quad coil in a horizontal bore 4.7 T magnet with Bruker console running ParaVision 5.1 software (Bruker BioSpin MRI GmbH, Ettlingen, Germany). SL was achieved using a *B*_1L_ pulse value of 90 μT (3.8 kHz), applied for 14 durations (TSL): 0.01, 0.05, 0.1, 0.5, 1.0, 5.0, 12.5, 25.0, 50.0, 75.0, 100.0, 125.0, 150.0, and 200.0 ms. This was followed by a rapid acquisition with relaxation enhancement (RARE) readout, using a coronal (horizontal) slice of thickness 1 mm. The acquisition parameters were: TR = 2000 ms; TE = 10 ms; field of view = 60 × 60 mm; matrix size = 128 × 128 pixels; RARE factor = 2; averages = 1; centric encoding. No spoiler gradients were applied between repetitions.

### Data Acquisition: *T*_1_ and *T*_2_

*T*_1_ and *T*_2_ values at 4.7 T were measured using a RARE saturation recovery imaging sequence without the preparatory SL pulse. Tubes were placed horizontally in the cradle and coil described previously and a single axial (vertical) slice used. The acquisition parameters for *T*_1_ were: recovery times = 57.2, 68.5, 78.5, 88.5, 103.5, 183.5, 283.5, and 383.5 ms; TE = 11 ms. For *T*_2_, TE ranged between 11 and 66 ms; TR (BSA) = 2000 ms; TR (PBS) = 8000 ms. Additional parameters common to all: field of view = 60 × 60 mm^2^; matrix size = 256 × 256 pixels; RARE factor = 2; averages = 1; centric encoding; slice thickness = 1 mm.

*T*_1_ and *T*_2_ measurements at 0.5 T were made on a Maran NMR spectrometer (Oxford Instruments, Abingdon, UK) utilizing a 20-MHz permanent magnet attached to a thermocouple heating mechanism and a PC running standard system software. *T*_1_ was measured using an inversion recovery sequence, with 20 log incremental inversion time recovery steps and 16 scans. TR was initially estimated for a test scan and then set to at least five times the expected final *T*_1_ value. *T*_2_ measurements were made using a standard Carr-Purcell-Meiboom-Gill sequence, with 1000 TE values.

### Model Fitting

On the images acquired at 4.7 T, circular regions of interest were drawn within each tube and the mean SI of each region of interest measured using ImageJ software (v1.42q, Rasband, W.S., ImageJ, U. S. National Institutes of Health, Bethesda, MD; http://imagej.nih.gov/ij/, 1997–2011). SI values were adjusted for noise bias using a simple Rician correction (23), based on mean standard deviations of four background regions in each image. Fitting of *R*_1ρ_ followed a nonlinear three-parameter fit suggested by Engelhardt and Johnson (24) using MATLAB (v7.9, MathWorks, Natick, MA) to determine the fully recovered SI (*S*_0_) values and relaxation rates (1/*T*_1ρ_), along with a parameter (*a*) to account for residual magnetization in the *y* axis due to the SL pulse (Eq. 1). *R*_1_ (1/*T*_1_) and *R*_2_ (1/*T*_2_) were determined using two-parameter nonlinear fits (Eqs. 2 and 3). For the inversion recovery data at 0.5 T, a three-parameter fit was applied to determine *S*_0_, *R*_1_, and *b*, a parameter accounting for imprecision in the 180° inversion pulse angle (Eq. 4).


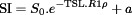
(1)


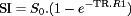
(2)


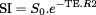
(3)


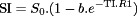
(4)

Confidence intervals were calculated at the 95% level. Datasets were compared for statistical significance at α = 0.05 using a paired *t*-test in SPSS (v 16.0, IBM SPSS, NY).

## RESULTS

**FIG. 1:**
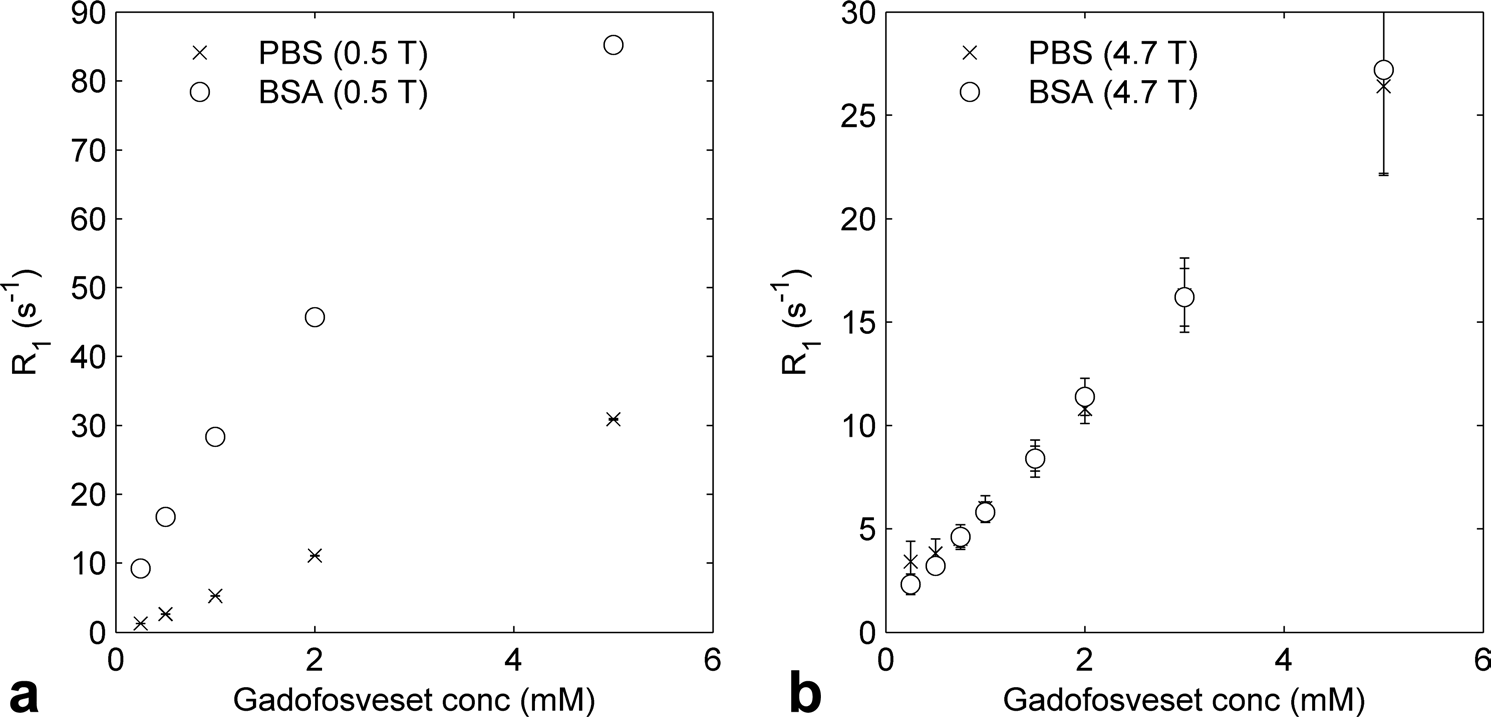
*R*_1_ values for gadofosveset in BSA (circles) and in PBS (crosses) at (**a**) 0.5 T and (**b**) 4.7 T. Error bars in (**a**) are smaller than data points.

**FIG. 2:**
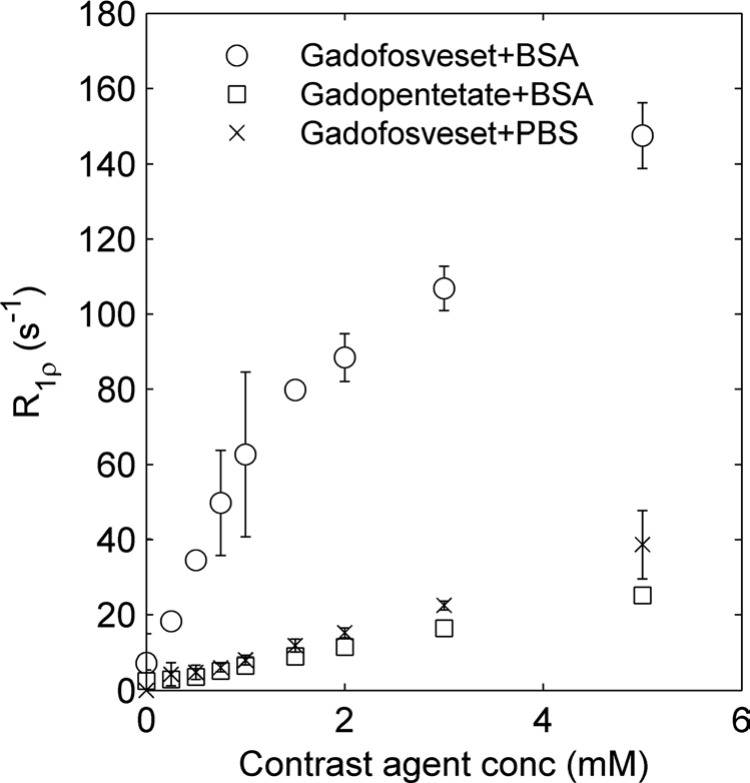
*R*_1ρ_ values for gadofosveset in BSA (circles) and in PBS (crosses) and gadopentetate in BSA (squares) at *B*_0_ = 4.7 T, *B*_1L_ = 90 μT.

**FIG. 3:**
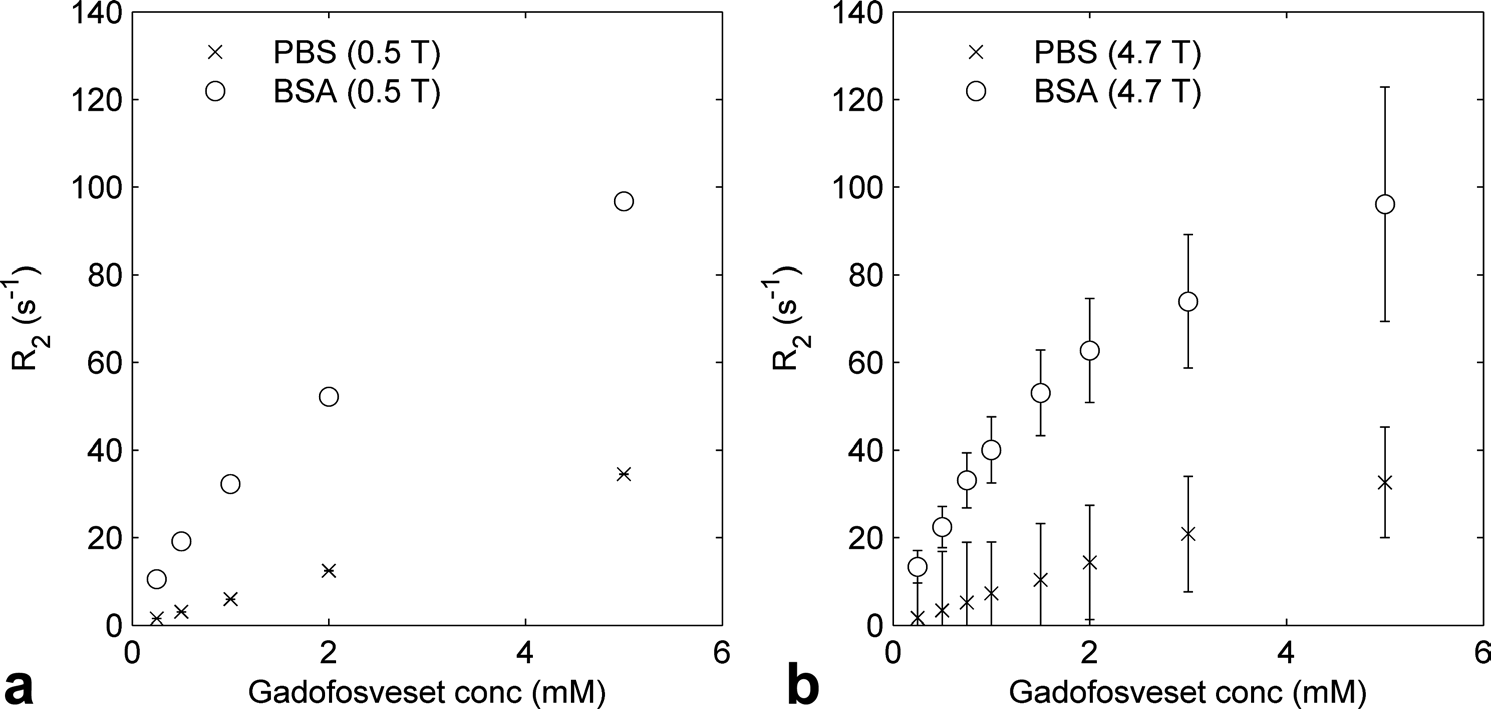
*R*_2_ values for gadofosveset in BSA (circles) and in PBS (crosses) at (**a**) 0.5 T and (**b**) 4.7 T. Error bars in (**a**) are smaller than data points.

Results are shown in Figs. 1–3, with error bars representing 95% confidence intervals. The overall *R*_1_ values for solutions of gadofosveset in BSA and in PBS were significantly different at *B*_0_ = 0.5 T (*P* = 0.003, Fig. 1a) but not different at 4.7 T (*P* = 0.757, Fig. 1b), confirming the lack of influence of binding on gadofosveset longitudinal relaxivity at high field strength. The *R*_1ρ_ relaxation rates for solutions of gadofosveset in BSA at 4.7 T were significantly higher than for gadofosveset in PBS (*P* = 0.001, Fig. 2). PBS *R*_1ρ_ values were similar to *R*_1_ values at 4.7 T (BSA and PBS solutions; Fig. 1b). The *R*_1ρ_ values for solutions of gadopentetate in BSA were similar to those for solutions of gadofosveset in PBS (*P* = 0.380, Fig. 2). *R*_2_ values for solutions of gadofosveset in BSA and in PBS at 0.5 T displayed a similar pattern to *R*_1_ values at this field strength, with significantly higher *R*_2_ values for the BSA solutions (*P* = 0.032, Fig. 3a). *R*_2_ values for equivalent solutions at 4.7 T were comparable with the *R*_2_ values at 0.5 T and the *R*_1ρ_ values at 4.7 T, with the BSA *R*_2_ values being significantly higher than the PBS *R*_2_ values (*P* < 0.001, Fig. 3b).

## DISCUSSION

The high binding fraction of gadofosveset to SA differentiates it from other clinically approved Gd-based contrast agents. The influence of binding on *R*_1_ is clear at 0.5 T (Fig. 1a) but is not observed at 4.7 T (Fig. 1b). Most clinical scanners operate at 3 T or lower, where an improvement in longitudinal relaxivity of gadofosveset over other agents is still observed (8). However, as clinical field strengths continue to increase, this advantage of high relaxivity diminishes and an alternative method for exploiting gadofosveset characteristics would be of benefit. This study demonstrates the feasibility of a previously unpublished method for combining the albumin-binding properties of gadofosveset with the macromolecular sensitivity of SL to generate improved contrast modification at high field strengths. *R*_1ρ_ values at 4.7 T for BSA solutions containing bound gadofosveset were found to be significantly greater than *R*_1ρ_ values for PBS solutions containing unbound gadofosveset at the same concentration (Fig. 2).

Because of the sensitivity of the SL technique to macromolecules, it is not clear from these findings alone the extent to which the difference in *R*_1ρ_ is attributable to the binding of gadofosveset or the presence of SA macromolecules. Comparison of *R*_1ρ_ values for BSA and PBS solutions in the absence of contrast agent (0 mM) should give an indication of the influence of the albumin molecules on SL relaxation. However, the lengthy relaxation times of these blank solutions led to poor model fits. Instead, the influence of albumin is better illustrated by measurements using the nonbinding contrast agent gadopentetate in BSA.

A previous study at contrast agent concentrations ≤ 0.5 mM (8) showed longitudinal relaxivity values at 4.7 T to be higher for gadofosveset in water than for gadopentetate in plasma (5.5 versus 3.7 mM^−1^ s^−1^, respectively). If the SL relaxivity of gadopentetate in BSA is found to be higher than that of gadofosveset in PBS, this may be attributable to the BSA solution macromolecules. The *R*_1ρ_ values for solutions of gadopentetate in BSA and gadofosveset in PBS (Fig. 2), and their associated relaxivity values, were not significantly different. The similarity of *R*_1ρ_ values for gadofosveset in PBS and gadopentetate in BSA together with the observation of relatively large *R*_1ρ_ values for gadofosveset in BSA all suggest, first, that the gadolinium has a greater effect on *R*_1ρ_ than the mere presence of the macromolecule, and second, that it is the binding rather than any nonspecific interactions with the protein that has the largest effect on *R*_1ρ_.

Although the SL contrast alteration observed with gadofosveset is not seen to the same extent with a small Gd-based nonbinding contrast agent in an equivalent macromolecular solution, SL has previously been successfully utilized in combination with nonbinding agents (14,17,19,20). The outcome of this study suggests that further benefit may be gained by exploiting the albumin-binding characteristics of an agent such as gadofosveset. In addition to increased *R*_1ρ_ relaxation rates, the kinetic behavior of gadofosveset is modified by binding, resulting in a lower extravasation rate and longer excretion time (2). Measurements of *R*_1_ at high field strength are unable to differentiate signal alteration from bound and free gadofosveset, due to their equivalent relaxivities. However, as *R*_1ρ_ is substantially altered by binding, it may be possible to use these measurements to differentiate bound and free gadofosveset at high fields.

It should be noted that for this in vitro study it was not necessary to optimize SL imaging parameters to take into account potential tissue heating issues resulting from high SAR. A relatively high *B*_1L_ value of 90 μT was chosen to give improved image quality; although *R*_1ρ_ increases as *B*_1L_ decreases, images become increasingly susceptible to artifacts caused by magnetic field inhomogeneities at very low *B*_1L_ values (12). Methods for reducing SAR, such as off-resonance SL (25), were not explored. To avoid SAR-related constraints when carrying out in vivo measurements, an alternative, more practical solution may be to exploit the differences between bound and free gadofosveset transverse relaxation rates. *T*_2_ values are routinely acquired on clinical scanners, and the effect of gadofosveset is clearly shown by *R*_2_ values in the presence and absence of albumin at 4.7 T (Fig. 3b). For these in vitro solutions, both *R*_2_ and *R*_1ρ_ demonstrate greater relaxivity for bound gadofosveset than *R*_1_. Although *T*_2_ measurement may be considered more practical, several studies, in particular those looking at tumor response to cytotoxic treatment, have suggested that *T*_1ρ_ may be a more responsive early indicator of physiological change than *T*_2_ (15, 16). It has also been suggested that improved (qualitative) tumor boundary definition may be achieved utilizing T_1ρ_ rather than *T*_2_ (26). A study of brain images in healthy volunteers at 1.5 T (27) found that *T*_1ρ_-weighted images displayed improved spatial resolution over *T*_2_-weighted images and *T*_1ρ_ maps had a greater dynamic range than equivalent *T*_2_ maps.

The scanning parameters at 4.7 T were optimized for physiological contrast agent concentrations. As a result, model fitting was less precise for the solutions containing the lowest and highest concentrations. The long *T*_1_ values on the 0 mM solutions caused particular problems with model fitting and were excluded from this analysis. In addition, the PBS *R*_2_ values at 4.7 T were based on a model fit to just three TE points, rather than the six points used for the BSA solutions, leading to greater imprecision in the calculated PBS *R*_2_ values.

For the purpose of this study, gadopentetate was assumed to be a nonbinding contrast agent, although there is some evidence to suggest that the chelate displays a weak tendency for binding to albumin (28). At the comparatively low SA concentration used here, however, the measured relaxation rates suggested little influence of albumin binding for gadopentetate and it may effectively be considered to be nonbinding.

In summary, this study has shown the *R*_1ρ_ response to gadofosveset in SA at high fields to be significantly larger than to a conventional small-molecule Gd-based contrast agent. This suggests that SL may be a viable method for regaining the *T*_1_ relaxivity lost by gadofosveset at high fields and may also provide an opportunity for additional tissue characterization through the differentiation of bound and free gadofosveset molecules. Despite offering potential benefits, implementation of this method in a SAR-limited clinical setting would require further investigation of optimal SL parameters prior to assessment in humans.
